# Targeted Next-Generation Sequencing in Uyghur Families with Non-Syndromic Sensorineural Hearing Loss

**DOI:** 10.1371/journal.pone.0127879

**Published:** 2015-05-26

**Authors:** Ying Chen, Zhentao Wang, Zhaoyan Wang, Dongye Chen, Yongchuan Chai, Xiuhong Pang, Lianhua Sun, Xiaowen Wang, Tao Yang, Hao Wu

**Affiliations:** 1 Department of Otorhinolaryngology-Head and Neck Surgery, Xinhua Hospital, Shanghai Jiaotong University School of Medicine, Shanghai, China; 2 Ear Institute, Shanghai Jiaotong University, Shanghai, China; 3 Shanghai Key Laboratory of Translational Medicine on Ear and Nose Diseases, Shanghai, China; Sant Joan de Déu Children's Hospital, SPAIN

## Abstract

The mutation spectrum of deafness genes may vary in different ethnical groups. In this study, we investigated the genetic etiology of nonsyndromic deafness in four consanguineous and two multiplex Uyghur families in which mutations in common deafness genes *GJB2*, *SLC26A4* and *MT-RNR*1 were excluded. Targeted next-generation sequencing of 97 deafness genes was performed in the probands of each family. Novel pathogenic mutations were identified in four probands including the p.L416R/p.A438T compound heterozygous mutations in *TMC1*, the homozygous p.V1880E mutation in *MYO7A*, c.1238delT frameshifting deletion in *PCDH15* and c.9690+1G>A splice site mutation in *MYO15A*. Co-segregation of the mutations and the deafness were confirmed within each family by Sanger sequencing. No pathogenic mutations were identified in one multiplex family and one consanguineous family. Our study provided a useful piece of information for the genetic etiology of deafness in Uyghurs.

## Introduction

Hereditary hearing loss is the most common neurosensory disorder in humans Among them, approximately 70% of cases are non-syndromic [1]. To date, more than 60 genes have been associated with non-syndromic deafness, including 55 autosomal recessive genes, 30 autosomal dominant genes, and 4 X-linked genes (http://hereditaryhearingloss.org/, updated in September 2014).

The genetic etiology of deafness may vary in different regions and races [2, 3]. In China, the mutation spectrum of deafness genes among Chinese Hans has been extensively studied. Bi-allelic *GJB2* mutations were reported in 19.1% of patients with non-syndromic deafness, followed by bi-allelic *SLC26A4* mutations in 12.1% and the mitochondrial *MT-RNR*1 mutations in 1.6% [2–4]. These three common deafness genes have been routinely screened in genetic testing of deafness. Furthermore, Our recent study showed that pathogenic mutations of rare deafness genes could be found in an additional 17.4% of Chinese Han deaf patients by targeted next-generation sequencing (NGS) of 79 deafness genes [5].

The molecular etiology study of deafness in other minorities in China, however, has been rare. Xinjiang, officially the Xinjiang Uyghur Autonomous Region, is the largest administrative division in China with about 10 million Uyghur habitants. In previous studies, nine hotspot mutations in *GJB2*, *SLC26A4*, *MT-RNR1* and *GJB3* were identified in 32.45% of Chinese Han deaf patients but only in 13.06% of Uyghur deaf patients in Xinjiang, indicating that those two ethnicities differed substantially in the mutation spectrum of the common deafness genes [6]. On the other side, mutations in the relatively rare deafness genes have not been studied in the Uyghur population. In the present study, we recruited four consanguineous and two multiplex recessive Uyghur families that were excluded from mutations in common deafness genes. Targeted next-generation sequencing of 97 deafness genes was performed in those six Uyghur families.

## Materials and Methods

### Subjects and clinical evaluations

**Fig 1:**
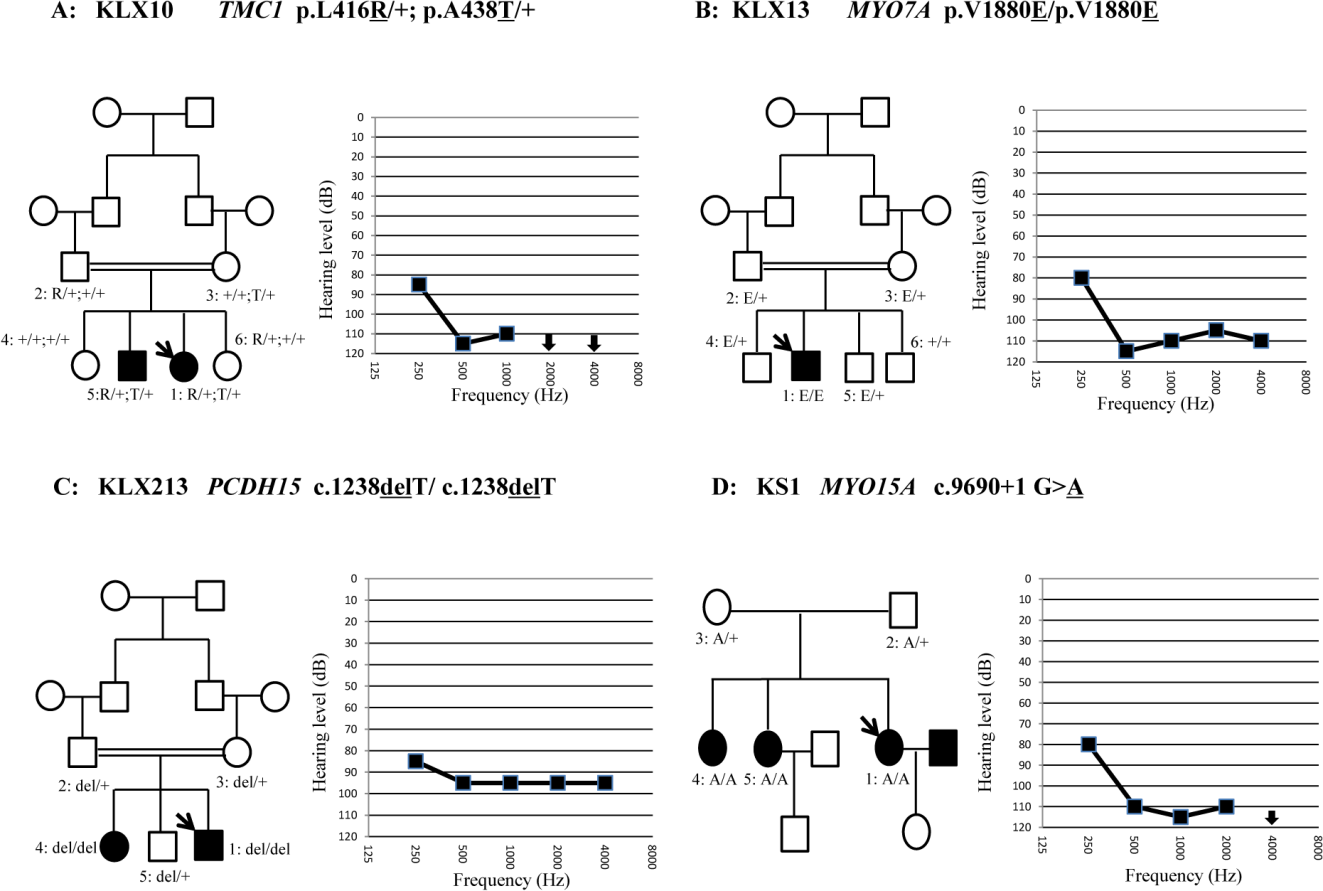
Pedigrees, genotypes and audiograms of Family KLX10 (A), KLX13 (B), KLX213 (C) and KS1 (D). Probands were pointed by an arrow.

**Fig 2:**
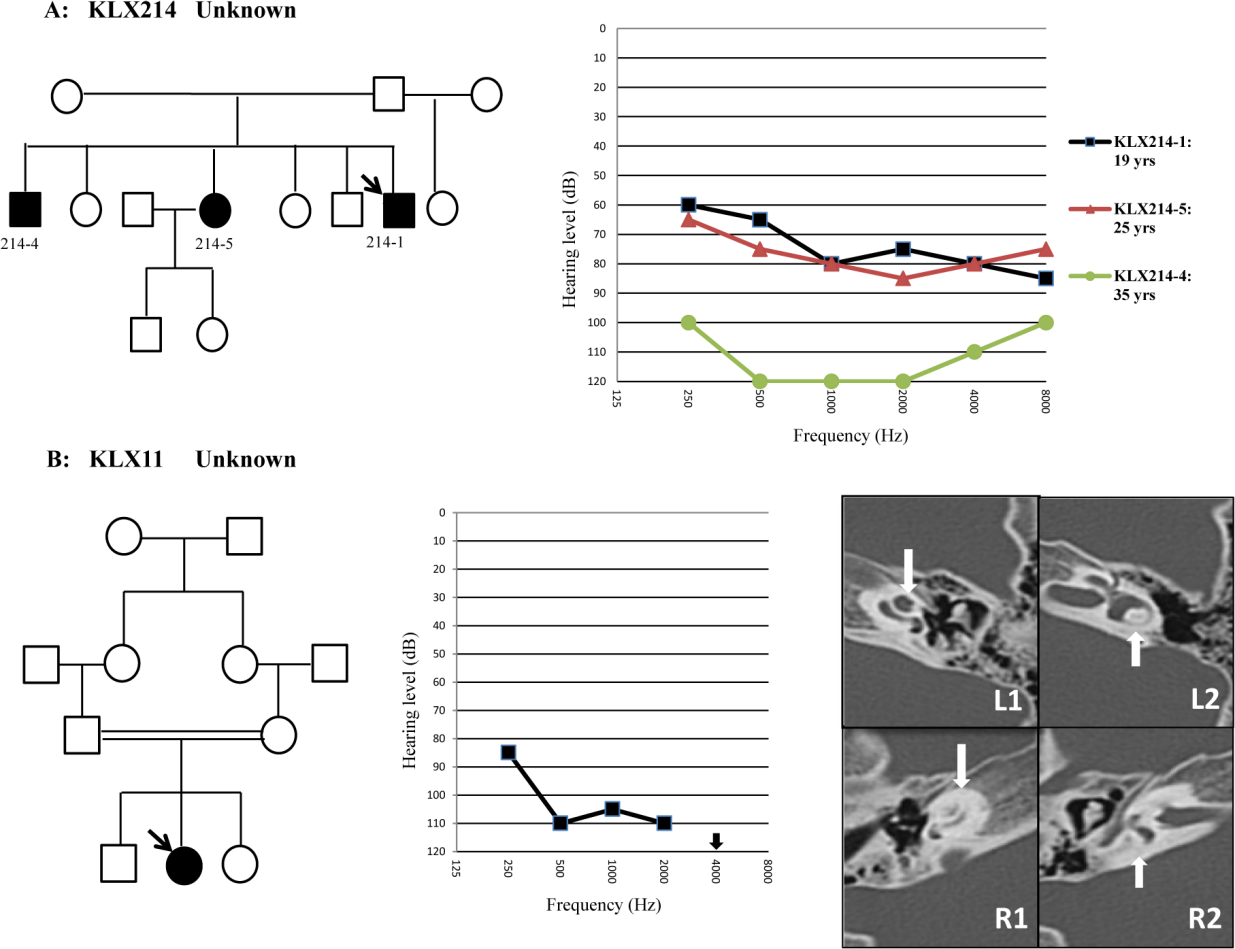
Pedigrees and audiograms of Family KLX214 (A) and KLX11 (B). Temporal bone CT scan of KLX11-1 showed dysplasia of the cochlea (downward arrow in R1) and the vestibule (upward arrow in R2) on the right side (R1, R2) in comparison with the normal inner ear structure on the left side (L1, L2).

Four consanguineous and two multiplex families were recruited from School for Deaf-Mutes of Kashgar, Xinjiang, China. The pedigrees of those six families are shown in Figs 1 and 2. All affected family members received a complete medical history inquiry and detailed physical examination to exclude the possibility of environmental causes or syndromic hearing impairment. Ophthalmologic examination including the eye fundus and visual field examination was performed for affected individuals KLX213-1, KLX213-4 and KLX13-1. Auditory evaluations were performed including otoscopic examination and pure tone audiometry. Degree of hearing impairment was calculated as the average of the hearing levels at 0.5, 1.0, 2.0 and 4.0 KHz for the better ear. The severity of hearing loss was defined as mild (20–40 dB), moderate (41–70 dB), severe (71–95 dB) and profound (>95 dB). Computed Tomography (CT) scan of the inner ear was performed in probands KLX214-1 and KLX11-1.

### Ethics statement

All subjects were recruited from School for Deaf-Mutes of Kashgar, Xinjiang, China from October 1, 2013 to December 31, 2013. They gave written, informed consent to participate in this study. This study was approved by the Ethics Committee of the Shanghai Jiaotong University School of Medicine, Xinhua Hospital and was in compliance with the Declaration of Helsinki.

### Mutation screening of common deafness genes

Genomic DNA was extracted from the blood samples using the Blood DNA kit (TIANGEN BIOTECH, Beijing, China). All probands were pre-screened against common deafness genes *GJB2*, *SLC26A4* and *MT-RNR1* by PCR amplification and bidirectional sequencing.

### Targeted next-generation sequencing

The amplified DNA was captured using biotinylated oligo-probes (MyGenostics, Beijing, China). The probes were designed to tile along all exons, splice sites and immediate flanking intron sequences of 97 deafness genes (S1 Table), including all known genes (at the time of this NSG panel was designed) involved in non-syndromic hearing loss and those involved in some relatively common syndromic hearing loss. Captured DNA fragments were sequenced on Illumina HiSeq2000 Analyzers. Data analysis and bioinformatics processing were performed following standard Illumina procedure. Reads were aligned to NCBI37/hg19 assembly using the BWA Multi-Vision software package. SNVs were detected and genotyped with the GATK UnifiedGenotyper in single-sample mode. Indels were identified using the GATK Indel Genotyper. Potentially pathogenic variants were defined as nonsense, missense, splice-site and indel variants that have allele frequencies under 0.01 (determined by databases including NCBI dbSNP, NHLBI ESP and 1000Genomes).

The candidate pathogenic mutations were genotyped by Sanger sequencing in all family members. Possible pathogenic effects of the missense mutations were evaluated by computational tools including Mutation Taster (http://www.mutationtaster.org), PROVEAN and SIFT (with cut-off scores set at -1.3 and 0.05, respectively, http://sift.jcvi.org). For CNV detection, the NGS data (including the in-house data from 200 Chinese Han normal hearing controls) were analyzed with the CNV calling tool CONTRA (Copy Number Analysis for Targeted Resequencing, http://contra-cnv.sourceforge.net/).

## Results

### Clinical characteristics of the Uyghur families

All affected members in the six Uyghur families had bilateral, prelingual, non-syndromic, severe-to-profound sensorineural deafness (Figs 1 and 2). CT scan showed that KLX11-1 had cochlear dysplasia: aplasia of the top turn and calcification of the middle and bottom turns. However, no record of meningitis was found in the patient’s history. Her hearing loss, however, was bilateral and symmetrical (Fig 1). The affected individuals KLX213-1, KLX213-4 and KLX13-1, who carried the homozygous mutations in *PCDH15* (KLX213-1 and KLX213-4) and *MYO7A* (KLX13-1), did not complain of any problems of visual acuity or visual field. Eye fundus examination of them showed no optic disc defects or retinal pigment degeneration.

### Identification of mutations by targeted NGS sequencing

Pre-screening of *GJB2*, *SLC26A4* and *MT-RNR1* identified no pathogenic mutations in the probands of the six families. Those probands were further screened by targeted NGS of 97 deafness genes. To identify the most likely pathogenic mutations, we filtered out: 1) all previously identified SNPs with allele frequencies of 0.005 or higher, 2) synonymous variants in the coding region and 3) variants in the intronic or untranslated regions (with the exception of the splice site mutations or variants that may create an ectopic splice site). A total of 1592 exons, 392354 bases were captured and sequenced in our study. The average depth for the targeted regions was 205.8-fold. 99.0% of the targeted regions were covered by 20 or more reads, demonstrating the high quality of the sequencing.

**Fig 3:**
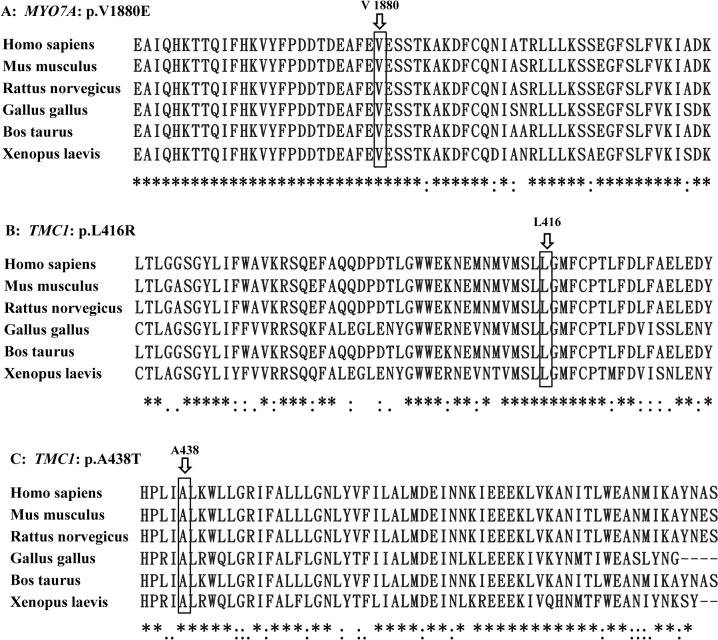
Multi-species sequence alignment showing the evolutionarily conserved residues of p.L416 and p.A438 in *TMC1* and p.V1880 in *MYO7A*.

Candidate variants were summarized in Supplementary Information (S2 Table). Consistent with the recessive inheritance of the Uyghur families, bi-allelic mutations were identified in four of the six probands, including the compound heterozygous mutations p.L416R/p.A438T of *TMC1* in KLX10-1, the homozygous p.V1880E mutation of *MYO7A* in KLX13-1, the homozygous c.1238delT mutation of PCDH15 in KLX213-1 and the homozygous c.9690+1G>A mutation of *MYO15A* in KS1-1. Genotyping of those mutations in the expanded family members showed that those mutations co-segregated with the deafness within the family (Fig 1). None of the mutations have been reported in previous studies or in the 1000-Genomes Project or the NHLBI Exome Sequencing Project. They were not present in 200 Chinese Han and 100 Uyghur normal hearing controls. The p.L416 and p.A438 residues of TMC1 and the p.V1880 residue of MYO7A were highly conserved (Phylop scores of 5.2, 6.3 and 4.8, respectively, Fig 3). The associated missense mutations were predicted to be disease causing by Mutation Taster (Table 1). The c.1238delT mutation of *PCDH15* was predicted to lead to a truncated protein product or nonsense-mediate decay of the mRNA. The c.9690+1G>A mutation in *MYO15A* was predicted to lead to aberrant splicing of the mRNA.

**Table 1:** Pathogenic mutations identified in the Uyghur families.

Family	Gene	Mutation type	Nucletide Change (Transcript version)	Amino acid change	Phylop Score^a^	Mutation Taster	PROVEAN (score)^b^	SIFT (score)^c^	Allele frequency in controls	Novelty
**KLX10**	*TMC1*	Missense	c.1247T>G (NM_138691)	p.L416R	5.242	DC^**d**^	Deleterious (-5.52)	Damaging (0.001)	0/600	novel
	*TMC1*	Missense	c.1312G>A (NM_138691)	p.A438T	6.306	DC	Neutral(-1.07)	Tolerated (0.699)	0/600	novel
**KLX13**	*MYO7A*	Missense	c.5639T>A (NM_001127180)	p.V1880E	4.835	DC	Deleterious (-5.41)	Damaging (0.000)	0/600	novel
**KLX213**	*PCDH15*	Frameshift	c.1238delT (OTTHUMT 00000291342)	p.M413RfsX8	-	-	-	-	0/600	novel
**KS1**	*MYO15A*	Splice site	c.9690+1G>A (NM_016239)	-	-	DC	-	-	0/600	novel

^a^Score ranges from -14 (not conserved) to 6.424 (conserved)

^b^Negative and positive scores indicate deleterious and neutral, respectively, with cut-off score set at -1.3

^c^Score ranges from 0 (deleterious) to 1 (neutral), with cut-off score set at 0.05

^d^DC = disease causing.

No pathogenic mutations were identified in Families KLX214 and KLX11. No potential pathogenic CNV (defined as present in the probands but not in 200 normal hearing controls) were identified by the NGS analysis.

## Discussion

Deaf children from consanguineously married families were relatively common in the Uyghur deaf population. Our previous study performed mutation screening of common deafness genes *GJB2*, *SLC26A4* and *MT-RNR1* in seven consanguineous Uyghur families and detected bi-allelic *SLC26A4* mutations in three of them [7]. The pathogenic mutations in the other four families, however, remained unclear.

Recent advances in targeted NGS provided a new strategy to identify mutations in deafness genes, especially in the relatively rare ones. Using this strategy, mutations in the rare deafness genes can be detected in between 23.2% and 62.5% of deaf patients that were excluded from mutations in *GJB2*, *SLC26A* or *MT-RNR1* [5, 8–10]. The mutation spectrum of the rare deafness genes was highly heterogeneous and variable in different ethnicities.

In this study, we performed targeted NGS in six Uyghur families including the aforementioned four consanguineous families and two additional multiplex recessive Uyghur families. All six families were excluded from mutations in *GJB2*, *SLC26A* or *MT-RNR1*. This is to our knowledge the first molecular etiology study of the rare deafness genes in Uyghurs. We indeed identified bi-allelic mutations in *TMC1*, *MYO7A*, *PCDH15* and *MYO15A* in four of the six families (Table 1 and Fig 1). Two homozygous mutations, c.1238delT in *PCDH15* and c.9690+1G>A in *MYO15A*, were truncating mutations that were highly likely to be pathogenic. The three missense mutations, compound heterozygous p.L416R/p.A438T in *TMC1* and homozygous p.V1880E in *MYO7A*, were likely to be pathogenic because: 1) these mutations segregated with the deafness in the family members; 2) p.L416R in *TMC1* and p.V1880E in *MYO7A* were predicted to be disease-causing by all three computational tools (Mutation Taster, PROVEAN and SIFT) while the p.A438T in *TMC1* was predicted to be disease-causing by Mutation Taster but neutral or tolerated by PROVEAN and SIFT; 3) all three mutations changed an evolutionarily conserved amino acid (Phylop scores≥4.8); 4) they were not seen in 200 Chinese Han and 100 Uyghur normal hearing controls or reported in the 1000-Genomes Project or the NHLBI Exome Sequencing Project; and 5) the prelingual, severe-to-profound hearing impairment observed in the corresponding family members was consistent with the auditory phenotype previously reported for recessive mutations in *TMC1* and *MYO7A* [11, 12].

In two of the four consanguineous Uyghur families, we identified homozygous mutations consistent with their consanguineous inheritance. Unexpectedly however, we identified compound heterozygous p.L416R/p.A438T mutations of *TMC1* in another consanguineous family KLX10, suggesting that the cause of deafness in this family was not originated from the consanguineous marriage. Similar cases have also been found in a consanguineous Uyghur family and a consanguineous Brazilian family, in which compound heterozygous mutations in *SLC26A4* and *MYO15A* were identified as the causes of hearing loss. [7, 13]

Mutations in PCDH15 and MYO7A may lead to both non-syndromic hearing loss (DFNB23 and DFNB2, respectively) and Usher syndrome type 1 (USH1F and USH1B, respectively). In this study, the affected individuals KLX213-1 and KLX213-4, who carried the c.1238delT homozygous mutation of PCDH15, and KLX13-1, who carried the p.V1880E homozygous mutation of MYO7A, were 17, 24 and 9 years old at the test, respectively. No ophthalmologic abnormalities were observed in any of them. Since the retinitis pigmentosa associated with Usher syndrome type 1 is usually evident within the first decade, we tentatively deemed that the associated hearing loss was non-syndromic.

Our targeted NGS of 97 deafness genes did not identify the causes of deafness in Families KLX214 and KLX11. Since those two families were multiplex (KLX214) or consanguineous (KLX11), they may harbor cryptic mutations that were undetectable by the current screening methods, such as copy number variants (CNVs), mutations in the non-coding regions or in the novel deafness genes. To test the former possibility, we performed a CNV analysis of the NGS data. No potential pathogenic CNV was identified. Interestingly, Family KLX214 showed a progressive pattern of hearing impairment (Fig 2), which was less commonly seen in recessive hearing loss. CT scan of KLX11-1, the only affected individual in the consanguineous family KLX11, detected unilateral cochlear dysplasia (Fig 2). By the CT scan, we couldn’t distinguish it was Mondini malformation or it resulted from occult meningitis. *SLC26A4* and *FGF3*, two genes that have been previously reported to be associated with deafness and Mondini malformation [14, 15], were included in our targeted NGS analysis. No pathogenic mutation of *SLC26A4* or *FGF3* was identified in KLX11-1. Further studies including the whole-exome sequencing may be needed to elucidate the causes of deafness in Families KLX214 and KLX11.

In summary, our targeted NGS analysis identified mutations of four rare deafness genes in four of the six (66.7%) Uyghur families, providing a useful piece of information for the genetic etiology of deafness in Uyghurs.

## Supporting Information

S1 Table Summary of the 97 targeted deafness genes. (DOC)

S2 Table All variants identified by targeted NGS. (DOC)
