# Supplementary material for: Targeted Next-Generation Sequencing in Uyghur Families with Non-Syndromic Sensorineural Hearing Loss
Source: PLoS One. 2015 May 26;10(5):e0127879. doi: 10.1371/journal.pone.0127879 (PMC4444116; doi:10.1371/journal.pone.0127879)
Supplement: S1 Table — (DOC) [file pone.0127879.s001.doc]

**S1 Table** . Summary of the 97 targeted deafness genes.

| **No** | **Gene** | **NS/S** | **Inheritance** | **Chromosome** | **Exons** | **Exon size (bp)** |
| --- | --- | --- | --- | --- | --- | --- |
| **1** | *ACTG1* | NS | AD | 17 | 7 | 1844 |
| **2** | *ALX3* | S | AR | 1 | 4 | 1482 |
| **3** | *CCDC50* | NS | AD | 3 | 12 | 8961 |
| **4** | *CDH23* | NS/S | AR | 10 | 68 | 11192 |
| **5** | *CHD7* | S(CHARGE） | AD | 8 | 38 | 11618 |
| **6** | *CLDN14* | NS | AR | 21 | 2 | 1753 |
| **7** | *CLRN1* | S(USH) | AR | 3 | 5 | 2554 |
| **8** | *COCH* | NS | AD | 14 | 11 | 2893 |
| **9** | *COL11A2* | NS/S | AR/AD | 6 | 64 | 6231 |
| **10** | *CRYM* | NS | AD | 16 | 9 | 1241 |
| **11** | *DFNA5* | NS | AD | 7 | 9 | 2274 |
| **12** | *DFNB31* | NS | AR | 9 | 12 | 2901 |
| **13** | *DFNB59* | NS | AR | 2 | 7 | 1531 |
| **14** | *DIAPH1* | NS | AD | 5 | 27 | 5790 |
| **15** | *DSPP* | NS | AD | 4 | 5 | 4336 |
| **16** | *ECM1* | S | AR | 1 | 9 | 1778 |
| **17** | *EDN3* | S(WS) | AR/AD | 20 | 5 | 2659 |
| **18** | *EDNRB* | S(WS) | AR/AD | 13 | 7 | 2537 |
| **19** | *ESPN* | NS | AR | 1 | 13 | 3544 |
| **20** | *ESRRB* | NS | AR | 14 | 11 | 3025 |
| **21** | *EYA1* | S(BOR) | AD | 8 | 16 | 3884 |
| **22** | *EYA4* | NS | AD | 6 | 19 | 5643 |
| **23** | *FGF3* | S | AR | 11 | 3 | 1551 |
| **24** | *FGF8* | S | AD | 10 | 5 | 861 |
| **25** | *FGFR1* | S | AD | 8 | 17 | 5645 |
| **26** | *FGFR3* | S | AD | 4 | 16 | 3967 |
| **27** | *FLNA* | S | XR | X | 47 | 8533 |
| **28** | *FOXI1* | S(PDS) | AR | 5 | 2 | 2298 |
| **29** | *FREM1* | S | AR | 9 | 14 | 4981 |
| **30** | *GATA3* | S | AD | 10 | 6 | 3073 |
| **31** | *GJB2* | NS | AR/AD | 13 | 2 | 2334 |
| **32** | *GJB3* | NS | AR/AD | 1 | 2 | 1764 |
| **33** | *GJB6* | NS | AR/AD | 13 | 3 | 1909 |
| **34** | *GPR98* | S(USH) | AR | 5 | 90 | 19340 |
| **35** | *GRHL2* | NS | AD | 8 | 16 | 5245 |
| **36** | *GRXCR1* | NS | AR | 4 | 4 | 996 |
| **37** | *HARS* | S | AR | 5 | 13 | 2219 |
| **38** | *HGF* | NS | AR | 7 | 18 | 2808 |
| **39** | *HMX1* | S | AR | 4 | 2 | 1898 |
| **40** | *HOXA2* | S | AD | 7 | 2 | 1780 |
| **41** | *IL13* | S | AR | 5 | 4 | 1282 |
| **42** | *KCNE1* | S(JLN) | AR | 21 | 2 | 3423 |
| **43** | *KCNQ1* | S(JLN) | AR | 11 | 16 | 3047 |
| **44** | *KCNQ4* | NS | AD | 1 | 13 | 3950 |
| **45** | *KRT9* | S | AD | 17 | 8 | 2297 |
| **46** | *LAMA3* | S | AR | 18 | 75 | 10580 |
| **47** | *LHFPL5* | NS | AR | 6 | 4 | 2151 |
| **48** | *LOXHD1* | NS | AR | 18 | 10 | 1850 |
| **49** | *LRTOMT* | NS | AR | 11 | 7 | 2832 |
| **50** | *MARVELD2* | NS | AR | 5 | 6 | 2130 |
| **51** | *MIR96* | NS | AD | / | / | 78 |
| **52** | *MITF* | S(WS) | AD | 3 | 9 | 4295 |
| **53** | *MYH14* | NS | AD | 19 | 41 | 6830 |
| **54** | *MYH9* | NS | AD | 22 | 41 | 7546 |
| **55** | *MYO15* | NS | AR | 17 | 66 | 11928 |
| **56** | *MYO15A* | NS | AR | 17 | 66 | 11928 |
| **57** | *MYO1A* | NS | AD | 12 | 28 | 3649 |
| **58** | *MYO3A* | NS | AR | 10 | 35 | 5819 |
| **59** | *MYO6* | NS | AR/AD | 6 | 35 | 8697 |
| **60** | *MYO7A* | NS/S | AR/AD | 11 | 27 | 4083 |
| **61** | *NF2* | S | AD | 22 | 15 | 5840 |
| **62** | *OTOA* | NS | AR | 16 | 19 | 2723 |
| **63** | *OTOF* | NS | AR | 2 | 29 | 4785 |
| **64** | *PABPN1* | S | AD | 14 | 7 | 3109 |
| **65** | *PAX3* | S(WS) | AD | 2 | 8 | 3118 |
| **66** | *PCDH15* | NS/S | AR | 10 | 35 | 5838 |
| **67** | *POU3F4* | NS | X-linked | X | 1 | 1508 |
| **68** | *POU4F3* | NS | AD | 5 | 2 | 1184 |
| **69** | *PROK2* | S | AR,AD | 3 | 3 | 1553 |
| **70** | *PROKR2* | S | AR,AD | 20 | 2 | 1157 |
| **71** | *PRPS1* | NS | X-linked | X | 4 | 1752 |
| **72** | *RDX* | NS | AR | 11 | 12 | 4131 |
| **73** | *RPGR* | S | XD | X | 19 | 3091 |
| **74** | *SALL1* | S | AD | 16 | 3 | 5256 |
| **75** | *SALL4* | S | AD | 20 | 4 | 3488 |
| **76** | *SEC23A* | S | AR | 14 | 20 | 3861 |
| **77** | *SIX1* | S(BOR) | AD | 14 | 2 | 2689 |
| **78** | *SIX5* | S(BOR) | AD | 19 | 3 | 3334 |
| **79** | *SLC17A8* | NS | AD | 12 | 11 | 3844 |
| **80** | *SLC26A4* | NS/S | AR | 7 | 21 | 4949 |
| **81** | *SLC26A5* | NS | AR | 7 | 10 | 1440 |
| **82** | *SNAI2* | S(WS) | AR | 8 | 3 | 2112 |
| **83** | *SOX10* | S(WS) | AD | 22 | 4 | 2866 |
| **84** | *STRC* | NS | AR | 15 | 29 | 5544 |
| **85** | *TCOF1* | S(TCO) | AD | 5 | 18 | 3880 |
| **86** | *TECTA* | NS | AR/AD | 11 | 23 | 6491 |
| **87** | *TIMM8A* | S | XR | X | 2 | 554 |
| **88** | *TMC1* | NS | AR/AD | 9 | 24 | 3225 |
| **89** | *TMIE* | NS | AR | 3 | 4 | 1865 |
| **90** | *TMPRSS3* | NS | AR | 21 | 13 | 3209 |
| **91** | *TPRN* | NS | AR | 9 | 4 | 2635 |
| **92** | *TRIOBP* | NS | AR | 22 | 8 | 1737 |
| **93** | *TRMU* | S | AR | 22 | 11 | 1975 |
| **94** | *USH1C* | NS/S | AR | 11 | 21 | 2258 |
| **95** | *USH1G* | S(USH) | AR | 17 | 3 | 3564 |
| **96** | *USH2A* | S(USH) | AR | 1 | 72 | 18955 |
| **97** | *WFS1* | NS | AD | 4 | 8 | 3644 |

NS: Non-Syndromic; S: Syndromic; AR: Autosomal Recessive; AD: Autosomal Dominant; BOR: Branchio-oto-renal syndrome; JLN: Jervell & Lange-Nielsen syndrome; STL: Stickler syndrome; TCO: Treacher Collins syndrome; USH: Usher syndrome; WS: Waardenburg syndrome
