# Supplementary material for: Targeted Next-Generation Sequencing in Uyghur Families with Non-Syndromic Sensorineural Hearing Loss
Source: PLoS One. 2015 May 26;10(5):e0127879. doi: 10.1371/journal.pone.0127879 (PMC4444116; doi:10.1371/journal.pone.0127879)
Supplement: S2 Table — (DOC) [file pone.0127879.s002.doc]

**S2 Table**. All variants identified by targeted NGS

| **Proband** | **Gene** | **Ref_number** | **Nucletide**  **change** | **Amino acid**  **change** | **Genotype** | **Allele frequency**  **in controls** | **Cosegragation with the deafness** |
| --- | --- | --- | --- | --- | --- | --- | --- |
| **KLX10-1** | *TMC1* | NM_138691 | c.1247T>G | p.L416R | Heterozygous | 0/600 | Yes |
|  |  |  | c.1312G>A | p.A438T | Heterozygous | 0/600 | Yes |
|  | *TCOF1* | NM_001195141 | c.3511C>T | p.P1171S | Heterozygous | 0/400 | No |
|  | *GPR98* | NM_032119 | c.18040T>C | p.F6014L | Heterozygous | 0/400 | No |
|  | *LRTOMT* | NM_001145308 | c.695C>T | p.A232V | Heterozygous | 0/400 | No |
| **KLX11-1** | *COL11A2* | NM_080680 | c.4547T>C | p.V1516A | Heterozygous | 0/400 | No |
|  | *DIAPH1* | NM_005219 | c.2200G>A | p.G734R | Heterozygous | 0/400 | No |
|  | *FLNA* | NM_001110556 | c.7039G>C | p.V2347L | Heterozygous | 0/400 | No |
|  | *LOXHD1* | NM_144612 | c.5023C>T | p.R1675C | Heterozygous | 0/400 | No |
|  | *OTOF* | NM_194248 | c.1117G>A | p.V373M | Heterozygous | 0/400 | No |
|  | *SALL1* | NM_002968 | c.3620G>A | p.G1207E | Heterozygous | 0/400 | No |
| **KLX13-1** | *MYO7A* | NM_001127180 | c.5639T>A | p.V1880E | Homozygous | 0/600 | Yes |
|  | *TRIOBP* | NM_001039141 | c.1589G>A | p.C530Y | Heterozygous | 0/400 | No |
|  |  |  | c.4508C>G | p.P1503R | Heterozygous | 0/400 | No |
| **KLX213-1** | *PCDH15* | ENST00000395442 | c.1238delT | p.M413RfsX8 | Homozygous | 0/600 | Yes |
| **KLX214-1** | *STRC* | NM_153700 | c.179T>C | p.F60S | Heterozygous | 0/400 | No |
|  | *LOXHD1* | NM_144612 | c.6463C>T | p.R2155C | Heterozygous | 0/400 | No |
|  | *TMIE* | NM_147196 | c.367_372del | p.123_124del | Heterozygous | 0/400 | No |
| **KS1-1** | *MYO15A* | NM_016239 | c.9690+1G>A | - | Homozygous | 0/600 | Yes |
|  | *CHD7* | NM_017780 | c.860C>T | p.P287L | Heterozygous | 0/400 | No |
